# Hydrogen Embrittlement Behavior of API X70 Linepipe Steel under Ex Situ and In Situ Hydrogen Charging

**DOI:** 10.3390/ma17194887

**Published:** 2024-10-05

**Authors:** Dong-Kyu Oh, Sang-Gyu Kim, Seung-Hyeok Shin, Byoungchul Hwang

**Affiliations:** Department of Materials Science and Engineering, Seoul National University of Science and Technology (SEOULTECH), 232 Gongneung-ro, Nowon-gu, Seoul 01811, Republic of Korea; 22532004@seoultech.ac.kr (D.-K.O.); rlatkdrb0323@seoultech.ac.kr (S.-G.K.); seunghyeok527@seoultech.ac.kr (S.-H.S.)

**Keywords:** linepipe steel, hydrogen embrittlement, electrochemical charging, slow strain-rate test, stress-oriented hydrogen-induced cracking (SOHIC)

## Abstract

This study investigates the hydrogen embrittlement behavior of API X70 linepipe steel. The microstructure was primarily composed of a dislocation-rich bainitic microstructure and polygonal ferrite. Slow strain-rate tests (SSRTs) were performed under both ex situ and in situ electrochemical hydrogen charging conditions to examine the difference between hydrogen diffusion and trapping behaviors. The ex situ SSRTs showed almost the same tensile properties as air and a limited brittle fracture confined to near the surface. In contrast, the in situ SSRTs showed an abrupt failure after the maximum tensile load, leading to a brittle fracture across the entire fracture surface with stress-oriented hydrogen-induced cracking (SOHIC). The crack trace analysis results indicated that SOHIC propagation paths were influenced by localized hydrogen accumulation due to high-stress fields. As a result, the dominant hydrogen embrittlement mechanisms, such as hydrogen-enhanced localized plasticity (HELP) and hydrogen-enhanced decohesion (HEDE), changed. These findings provide critical insights into the microstructural factors affecting hydrogen embrittlement, which are essential for the design of hydrogen-resistant steels in hydrogen infrastructure applications.

## 1. Introduction

As hydrogen gains attention as an eco-friendly energy source, developing reliable infrastructure for its transportation has become a critical challenge for the commercialization of the hydrogen economy [1,2]. Various grades of American Petroleum Institute (API) linepipe steels, consisting of bainitic microstructures, have been used not only for transporting natural gas but also for high-pressure gaseous hydrogen transportation [3,4]. However, sour components from natural gas or gaseous hydrogen can cause hydrogen embrittlement in various ways [5], such as blistering, hydrogen-induced cracking (HIC) [6], and stress-oriented hydrogen-induced cracking (SOHIC) [7,8]. Hydrogen atoms penetrate the specimen from the surface, interacting with microstructural defects such as vacancies, dislocations, and grain boundaries and leading to hydrogen-related degradation of mechanical properties [9]. These interactions influence the mechanical properties and contribute to crack initiation and propagation. Understanding these hydrogen embrittlement phenomena is important for designing microstructures with good hydrogen embrittlement resistance to ensure the integrity of hydrogen structural components.

The hydrogen embrittlement of carbon steels has been studied for decades [10,11]. Generally, hydrogen embrittlement is investigated by conducting a slow strain-rate test to ensure sufficient time for hydrogen to diffuse and interact with microstructural defects [12,13]. Various tests have been conducted, considering factors such as stress state, hydrogen source, microstructural factors, and hydrogen charging method. While classical methods with varying hydrogen charging conditions have been widely used to study hydrogen embrittlement, more advanced techniques like atomic probe tomography (APT) and in situ transmission electron microscopy (TEM) have also been employed in recent years [14,15]. However, the precise mechanisms of hydrogen embrittlement remain unclear. Although the exact mechanisms of hydrogen embrittlement are not fully understood, there is clear evidence that hydrogen embrittlement occurs, leading to degradation in the mechanical properties of API linepipe steels [16,17]. Specifically, both ex situ and in situ hydrogen charging methods have been employed to study hydrogen embrittlement, resulting in different levels of hydrogen-related degradation of mechanical properties. The ex situ hydrogen charging method is straightforward and convenient. However, hydrogen atoms are only located on specimen surfaces and diffuse into the air during the test, which reduces notable hydrogen embrittlement phenomena [18]. In contrast, the in situ hydrogen charging method ensures sufficient hydrogen supply to the specimen during testing, thus avoiding the drawbacks associated with the ex situ hydrogen charging method. The characteristics of these methods must be carefully considered, particularly due to the small atomic radius of hydrogen, which facilitates its diffusion in the lattice sites of steels. Additionally, the bainitic steels in linepipe and martensitic steels in hydrogen high-pressure vessels have a body-centered structure with wide lattice sites, allowing for easy diffusion. As a result, these steels exhibit similar fracture behaviors, such as cleavage fractures and tearing in hydrogen conditions, though the extent of embrittlement can vary significantly depending on the testing method [19].

For these reasons, comparative studies between in situ and ex situ methods have become more common in investigating the effects of external and internal hydrogen [20]. Internal hydrogen refers to pre-charged hydrogen within the specimen, representing the ex situ hydrogen charging condition. In contrast, external hydrogen refers to hydrogen introduced into the specimen from the environment, which is represented by the in situ hydrogen charging condition. Although research on hydrogen embrittlement under ex situ and in situ hydrogen charging is becoming more prevalent, there is still a lack of comprehensive studies that clarify the correlation between microstructural features and the fracture behaviors deriving from the mechanism of hydrogen.

In this paper, slow strain-rate tests (SSRTs) were conducted on API X70 linepipe steel under both ex situ and in situ electrochemical hydrogen charging conditions to discuss the fracture behavior transition based on hydrogen diffusion and trapping behaviors. The crack trace technique was also employed to understand the correlation between the mechanisms of hydrogen embrittlement and microstructural factors by investigating the SOHIC propagation path.

## 2. Materials and Methods

The chemical composition of the API X70 linepipe steel was Fe-0.05C-1.1Mn-0.16Si-0.17Cr (wt.%). The steel used in this study was produced through a thermo-mechanical controlled process (TMCP). The initial slab was reheated to 1200 °C. After reheating, controlled rolling was conducted until the thickness was reduced to 9.2 mm, with a finishing temperature of 830 °C. Following the controlled rolling, accelerated cooling was applied at a cooling rate of 30 °C/s until the steel plate reached a temperature below 600 °C. After the accelerated cooling process, the API X70 steel plate was coiled. Microstructural analysis was conducted on the 1/4 thickness region of the longitudinal–transverse (L-T) plane of the steel. 

The specimen used to observe the microstructure was polished and etched with a 3% Nital etchant in 15 to 30 s, and it was examined using a scanning electron microscope (SEM, EVO10 Carl Zeiss, Jena, Germany). Additionally, electron back-scattered diffraction (EBSD, Symmetry S2, Oxford Instruments, Oxford, UK) analysis was performed to examine the microstructure. The acceleration voltage, working distance, and step size for the EBSD analysis were 15 kV, 11 mm, and 0.18 μm, respectively. Based on the grain orientation spread (GOS) map of EBSD results, grains with a GOS level below 2° were considered fully recrystallized ferrite, while the remaining microstructures were identified as bainitic microstructures, formed during accelerated cooling.

Slow strain-rate tests (SSRTs) were performed at a crosshead speed of 0.075 mm/min using a 10-ton universal testing machine (UT-100E, MTDI, Daejeon, Republic of Korea) with sub-sized plate-type tensile specimens having a gauge width of 6.3 mm, a thickness of 2.0 mm, and a gauge length of 25.0 mm according to the ASTM E8 standard testing method [21]. The selected crosshead speed corresponds to a strain rate of approximately 5 × 10^−5^/s, which falls within the range recommended by ASTM G129 for slow strain-rate tests [22]. The electrochemical hydrogen charging current density under both ex situ and in situ SSRTs was 20 A/m^2^, and the electrolyte was a 1 M NaOH + 3 g/L NH_4_SCN aqueous solution. The ex situ SSRT specimens were pre-charged for 24 h. For the ex situ SSRT, specimens were pre-charged with hydrogen for 24 h prior to testing to ensure the specimens were saturated with hydrogen. In contrast, during the in situ SSRT, hydrogen charging was continuously applied throughout the entire test.

Crack trace analysis was conducted based on EBSD data of the cracks on the side surface of the in situ SSRT specimen. The pole figures of the two grains adjacent to the crack path were examined, and if their poles were nearly identical, it was concluded that the two grains originated from a single grain before the fracture. The crystallographic orientation and crack propagation direction were then correlated using the pole figure data. Finally, the nearest slip or cleavage plane was identified to determine the crystallographic pathway through which the crack propagated.

## 3. Results and Discussion

### 3.1. Microstructure and Mechanical Behavior under Different Hydrogen Charging Conditions

**Figure 1:**
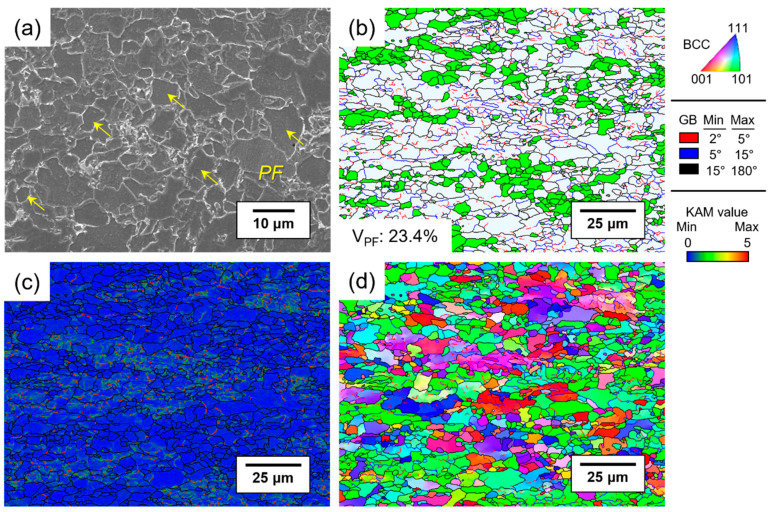
(**a**) Scanning electron microscope (SEM) images showing the polygonal ferrite with yellow arrows; (**b**) grain orientation spread (GOS) map highlighting polygonal ferrite in green based on a GOS level of under 2° and surrounded by high-angle grain boundaries; (**c**) kernel average misorientation (KAM) map; and (**d**) inverse pole figure (IPF) map from the results of electron back-scattered diffraction (EBSD) analysis.

Figure 1 provides SEM images and EBSD analysis results of the microstructure of API X70 steel. Polygonal ferrite (PF) and bainitic microstructures with complex morphologies were observed in the API X70 steel (Figure 1a). Figure 1b shows the grain orientation spread (GOS) map highlighting PF grains in green with a volume fraction of 23.4%. These grains are characterized by a GOS level under 2°, surrounded by high-angle grain boundaries with equiaxed morphology [23]. The kernel average misorientation (KAM) and inverse pole figure (IPF) maps indicate that grains, except for polygonal ferrite, had a high localized KAM level and color gradient due to substructure and high dislocation density (Figure 1c,d). Specifically, the fine, acicular-shaped regions with high KAM levels correspond to acicular ferrite, while the coarse grains with low-angle boundaries within them are identified as granular bainite. These grains are identified as bainitic microstructures, which are the dominant factors affecting hydrogen embrittlement behavior in this study due to their high volume fraction of over 75% [24].

**Figure 2:**
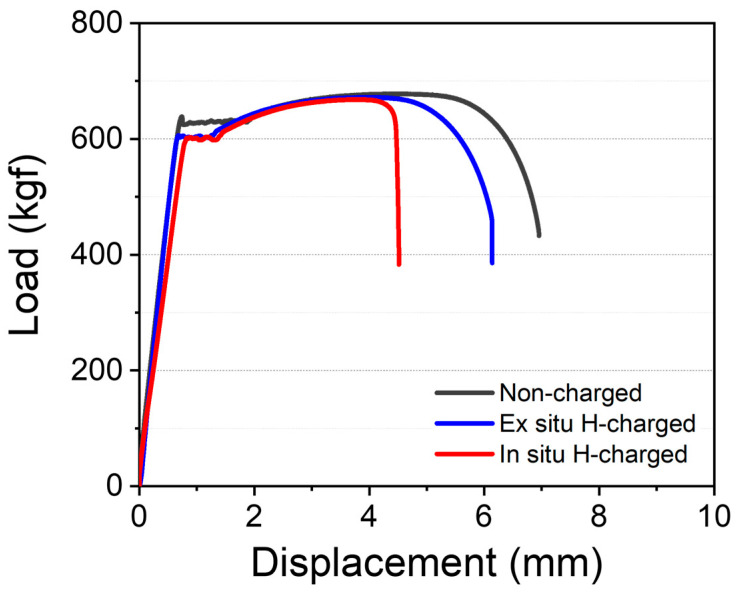
Load–displacement curves of slow strain-rate tests (SSRTs) under non-charged, ex situ, and in situ hydrogen charging conditions with a current density of 20 A/m^2^.

Figure 2 shows the load–displacement curves obtained from the non-charged, ex situ, and in situ SSRTs. As shown in the load–displacement curve, the maximum tensile load of steel was almost the same. However, the ex situ SSRT results show less displacement loss compared to those of the in situ SSRT results. This is because during the pre-charging and ex situ SSRT procedures, hydrogen was located only on the specimen surfaces, and it diffused into the air, affecting only the early stage of the SSRTs due to the high hydrogen diffusion coefficient of the body-centered cubic (BCC) lattice [25,26,27,28]. Meanwhile, in the case of the in situ SSRT, hydrogen atoms were continuously introduced into the specimen due to the cathodic reaction during testing, resulting in abrupt failure after the maximum tensile load.

### 3.2. Fractographic Analysis

**Figure 3:**
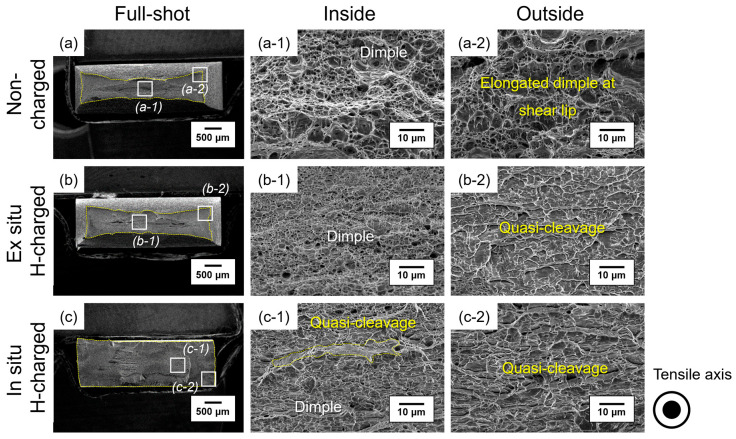
Secondary electron microscope (SEM) fractographs of slow strain-rate tested specimens (**a**,**a-1**,**a-2**) in air, (**b**,**b-1**,**b-2**) under ex situ hydrogen charging, and (**c**,**c-1**,**c-2**) under in situ hydrogen charging, with a current density of 20 A/m^2^. The inside and outside of the fracture surface were observed in the magnified image with identifying fracture mode.

As shown in Figure 3a, non-charged specimens exhibited a typical ductile fracture with fine dimples appearing throughout the gauge section of the specimen. Fractography of the inside and outside regions showed equiaxed dimples and elongated dimples, along with the presence of shear lips caused by necking. For the ex situ SSRT specimens, the inside region also exhibited a ductile fracture mode, similar to the non-charged SSRT specimens, while the outside region indicated a quasi-cleavage fracture mode (Figure 3b). Quasi-cleavage fracture is a type of brittle fracture that occurs within the grains, and it is characterized not only by fracture along the cleavage plane but also by a tearing ridge formation along the slip system due to localized plastic deformation. In some cases, quasi-cleavage can include regions where dimples are not fully developed, or it can even coexist with dimples, as observed in this study. As seen in the slight displacement loss in the ex situ SSRT results (Figure 2), pre-charged hydrogen was located very close to the surface, and it diffused into the air during testing, dramatically changing the fracture mode only in the outside region [29]. This phenomenon, where the transition to a brittle fracture mode occurs in the presence of hydrogen, is reported to happen only when hydrogen exceeds a specific concentration, known as the critical hydrogen concentration [30,31].

Meanwhile, as shown in Figure 3c, the in situ SSRT specimen exhibited a quasi-cleavage fracture mode without necking, regardless of the distance from the surface of the tested specimen. Unlike in the ex situ SSRT, where hydrogen concentration reached a critical level only on the surface, the in situ SSRT involved simultaneously supplying fresh hydrogen into the specimen. Therefore, hydrogen diffused deeper and maintained a hydrogen concentration above the critical value more easily during testing compared to the ex situ SSRT, resulting in a quasi-cleavage fracture across the entire fracture surface.

### 3.3. Stress-Oriented Hydrogen-Induced Cracking (SOHIC)

**Figure 4:**
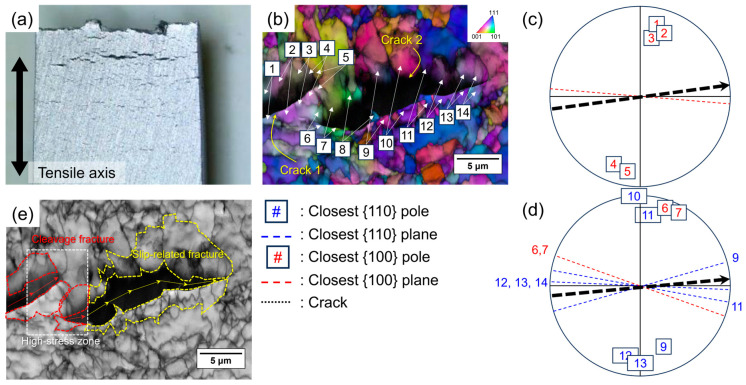
(**a**) Macro images of stereographic microscopy of stress-oriented hydrogen-induced cracking (SOHIC); (**b**) inverse pole figure (IPF) maps notated with numbers corresponding to the analyzed grains by crack trace technique; (**c**,**d**) stereographic projection with the position of the closest cleavage plane and slip system for the numbers (#) corresponding to the grains indicated in (**b**) to the crack propagation path; (**e**) band contrast map highlighting fracture behavior and the high-stress zone.

Figure 4a shows many cracks perpendicular to the tensile axis, found only on the surface of the gauge part in the in situ SSRT specimen. These cracks act as hydrogen penetration paths into the specimen during testing, causing a transition in fracture behavior from ductile to brittle even on the inside of the specimen. The plastic deformation along the tensile axis was relieved by the introduction of new surfaces from the cracks on the surface of the gauge part, rather than from the necking—as in the ex situ SSRT specimen. In this study, both ductile and brittle fractures occurred, and fracture behavior changed when the regions with a hydrogen concentration higher than the critical level generated cracks [32]. These perpendicular cracks are widely known as stress-oriented hydrogen-induced cracking (SOHIC) and are closely related to hydrogen embrittlement phenomena, which involve a transition in fracture behavior (Figure 4a) [33]. Generally, SOHIC occurs when applied stress and hydrogen charging happen simultaneously. This is because hydrogen accumulates in the high-stress field at vulnerable spots, resulting in cracks that are perpendicular to the tensile axis.

To understand how the microstructure affects the propagation path of SOHIC, a crack trace technique was used in conjunction with the EBSD results. Figure 4b shows the IPF map with each fractured grain numbered to identify their crystallographic orientations. The stereographic projections (Figure 4c,d) show the position of the cleavage plane and slip system closest to the crack trace in the identified fracture grains for each crack. The SOHIC mainly showed transgranular fractures, with very few cases of intergranular fractures (grain 8). Transgranular fractured grains exhibited different fracture behaviors depending on their crystallographic orientation. Specifically, the SOHIC in grains 1 to 7 propagated through the {100} cleavage planes, while grains 9 to 14 showed slip-related fractures. This difference in crystallographic orientation for the SOHIC propagation path suggests that the hydrogen embrittlement mechanism is influenced by microstructural factors and the applied stress state.

To date, various hydrogen embrittlement mechanisms have been proposed, such as hydrogen-enhanced decohesion (HEDE) and hydrogen-enhanced localized plasticity (HELP) [34]. These mechanisms are based on the observation that hydrogen-related degradation in metals strongly depends on defects that act as hydrogen trap sites, such as lattice sites, dislocations, and grain boundaries [35].

The API X70 steel used in this study has a dislocation-rich bainitic microstructure matrix and a polygonal ferrite volume fraction of 23.4%. Therefore, the hydrogen atoms mostly trapped in dislocations—causing SOHIC propagation along the slip plane—are a major factor in hydrogen embrittlement [36,37]. Hydrogen enhances the mobility of dislocations due to its shielding effect on the dislocation elastic field [32], resulting in localized plastic flow with crack initiation and propagation according to the HELP mechanism. However, the crack trace analysis for SOHIC showed different crack propagation based on crystallographic orientation between grains 1 to 7 and grains 9 to 14, despite the same direction on the macro-scale (Figure 4e).

**Figure 5:**
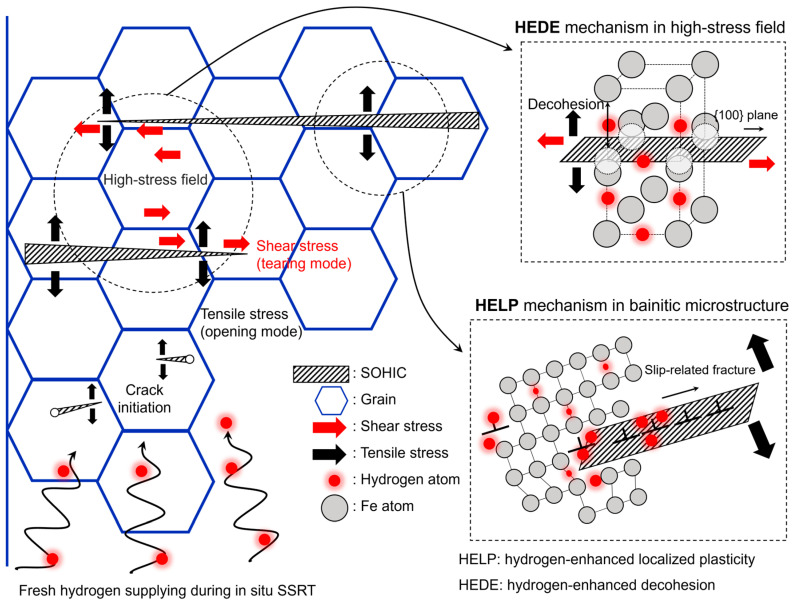
Schematic illustration of stress-oriented hydrogen-induced cracking (SOHIC) propagation mechanisms during in situ slow strain-rate test (SSRT).

As mentioned above, the BCC lattice has a high hydrogen diffusion coefficient due to the large spaces in interstitial sites [23,24,25]. Hydrogen atoms are preferentially trapped at dislocations, as well as at tetrahedral or octahedral sites in the BCC lattice, reducing the cohesive energy and atomic bonding force, which then leads to cleavage or intergranular fracture. Additionally, the difference in crack propagation paths can be related to the stress state ahead of the crack tips and the interaction area between cracks. In the region of grains 1 to 7, the stress field is identified by mixed-mode (opening and shearing) loading caused by crack interaction, and the spatial distribution of the maximum shear and tensile stresses is applied between the cracks [38,39]. As a result, grains 1 to 7 fractured along the {100} cleavage planes due to high localized stress at the intersection between cracks and the HEDE mechanism caused by hydrogen trapped at interstitial sites. Figure 5 provides the schematic illustration of SOHIC propagation mechanisms described above. These results reveal that a local hydrogen concentration higher than the critical level was achieved only locally in the microstructure due to stress concentration and stress-driven hydrogen accumulation at the intersection of cracks, resulting in a cleavage fracture along the {100} planes.

## 4. Conclusions

This study provides a comprehensive analysis of the hydrogen embrittlement behavior of API X70 linepipe steel, which predominantly consists of a bainitic microstructure with a high dislocation density and a moderate fraction of polygonal ferrite. The results from slow strain-rate tests (SSRTs) under both ex situ and in situ hydrogen charging conditions reveal distinct fracture behaviors influenced by hydrogen diffusion and trapping. In situ hydrogen charging caused more extensive embrittlement with stress-oriented hydrogen-induced cracking (SOHIC), while ex situ embrittlement was limited to the surface. SOHIC propagation depended on local stress fields and microstructural interactions with hydrogen. HELP dominated crack propagation, while HEDE occurred at crack intersections with higher localized stresses. These findings provide valuable insights into the hydrogen embrittlement of ferritic linepipe steels used for high-pressure hydrogen transport, offering improved strategies for microstructural design and assessment.

## Data Availability

Data are contained within the article.
